# Analysis and modeling of coolants and coolers for specimen transportation

**DOI:** 10.1371/journal.pone.0231093

**Published:** 2020-04-17

**Authors:** David E. Lowe, Gerald Pellegrini, Elizabeth LeMasters, Andrew J. Carter, Zachary P. Weiner

**Affiliations:** 1 National Center for Emerging and Zoonotic Infectious Diseases, Centers for Disease Control and Prevention, Atlanta, GA, United States of America; 2 Laboratory Leadership Service Fellowship, Centers for Disease Control and Prevention, Atlanta, GA, United States of America; 3 Center for Global Health, Centers for Disease Control and Prevention, Atlanta, GA, United States of America; Copenhagen University Hospital Holbæk, DENMARK

## Abstract

Maintaining cold chain while transporting medical supplies and samples is difficult in remote settings. Failure to maintain temperature requirements can lead to degraded sample quality and inaccuracies in sample analysis. We performed a systematic analysis on different types of transport coolers (polystyrene foam, injection-molded, and rotational molded) and transport coolants (ice, cold packs, frozen water bottles) frequently in use in many countries. Polystyrene foam coolers stayed below our temperature threshold (6°C) longer than almost all other types of coolers, but were not durable. Injection-molded coolers were durable, but warmed to 6°C the quickest. Rotational molded coolers were able to keep temperatures below our threshold for 24 hours longer than injection molded coolers and were highly durable. Coolant systems were evaluated in terms of cost and their ability to maintain cold temperatures. Long lasting commercial cold packs were found to be less cost effective and were below freezing for the majority of the testing period. Frozen plastic water bottles were found to be a reusable and economical choice for coolant and were only below freezing briefly. Finally, we modeled the coolers performance at maintaining internal temperatures below 6°C and built a highly accurate linear model to predict how long a cooler will remain below 6°C. We believe this data may be useful in the planning and design of specimen transportation systems in the field, particularly in remote or resource limited settings.

## Introduction

Accurate results from public health laboratories are dependent on specimens arriving in good condition. Many specimens shipped from the site of collection to the testing laboratory facility must be maintained at refrigeration temperatures (between 2° and 8°C) to ensure specimen stability. The process of keeping samples cold during transit is known as the specimen cold chain. Disrupting the cold chain during specimen transport activities can lead to specimen degradation and decrease the accuracy of testing or therapeutic potency [1–4]. Cold chain can be difficult to maintain during long trips or when traveling in areas with under-developed infrastructure [5–7]. Therefore, improving cold chain can decrease the number of specimens or medical supplies that arrives unfit for use.

During transportation, biological samples and laboratory reagents are placed in insulated coolers to extend cold chain times. Several high priced options exist that can keep specimens cold for extended periods of time; however, these may not be feasible or available for low resource countries. In these settings, understanding the capabilities and limitations of less expensive coolers and coolants can help make data-driven decisions on their cold chain system. These coolers are typically a polystyrene (PS) foam container in a cardboard box or plastic injection-molded coolers. Both coolers are relatively inexpensive to purchase but there are limitations. PS foam is thought to keep items cold for longer periods of time than injection-molded coolers, but they are less durable and difficult to decontaminate for reuse. As such, PS foam coolers are discarded after use, making it a poor choice in sustaining specimen transport networks in low resource environments with procurement challenges. Plastic injection molded coolers have non-porous surfaces which allows them to be decontaminated between uses. These coolers are made by injecting molten plastic into a mold, allowing for rapid production and lower cost. However, this process limits the thickness of the plastic and can produce flaws in the plastic that lead to reduced durability. Injection molded coolers have thinner insulated walls that do not maintain cold temperatures as long as PS foam cooler. A newer type of plastic cooler, rotational molded (from here on called rotomolded) coolers appear to offer both the advantage of prolonged cold chain and reusability. To increase durability, a different process is used to create a uniform thick layer of plastic. Rotomolded cooler construction involves melting plastic beads in a mold as it rotates vertically and horizontally, allowing even and thicker coating of the mold, for a more durable layer of plastic. They also have thicker amounts of insulation and feature gasket sealed lids. Finally, most of these coolers can be locked. However, these coolers are less affordable than injection molded and PS foam coolers. Yet, there is little peer-reviewed literature comparing how these coolers can prolong cold chain stability in specimen transport operations.

The type and amount of coolant material used within the cooler during specimen transfers is another major factor affecting the cold chain system. Replenishing coolant material in low resource environments can be challenging due to unreliable electrical power (to support refrigerators and freezer units) and longer transit times due to poor road infrastructure transportation networks [5, 6, 8, 9]. Therefore, it is necessary to find coolants that can cool for long transport times. Often times, frozen cold packs are used in lieu of ice because they are reusable, seldom leak and provide colder temperatures than ice as they thaw. An alternative to reusable cold packs and ice are frozen water bottles. Plastic water bottles are readily available in even the most remote, low resource regions and are inexpensive to purchase. Further, many cold packs will hold temperatures below 0°C, which can damage certain types of specimens [10]. But ice and frozen water bottles will always stabilize at 0°C. There are multiple cold packs available at a range of costs, but it is not well understood how they compare to one another or economical alternatives such as a frozen water bottles.

To understand how coolers and their coolants affect cold chain, we tested the ability of various coolers and coolants to maintain temperatures between 0 and 6°C. PS foam, injection molded and rotomolded coolers were compared against each other for their ability to maintain cold temperatures. Various cold packs were also evaluated for their ability to retain temperatures below 6°C. For both the coolers and coolant packs, performance was compared along with purchase price. In addition, we used different amounts of frozen water bottles to build a predictive model in all three cooler types. This model gives an accurate prediction of how long a cooler will remain below 6°C based on the amount of coolant added. Our study provides a point of comparison between differing coolers and coolants and a predictive model to provide data to laboratory staff and epidemiologists to use in specimen transportation planning.

## Materials and methods

### Coolers and coolants

Coolers and coolants were purchased from either Amazon.com or the cooler company’s website. Prices were determined by the cost of the item at the time of purchase in 2017, excluding taxes and shipping. Coolers were de-identified and assigned an arbitrary study number. All coolers had an internal volume between 8.7 and 29.2 liters. Coolant packs were all approximately 0.45 kg per cold pack. Frozen water bottles were made by filling 0.5 L plastic bottles with 0.45 kg of deionized water. All coolants were placed in a -20°C freezer overnight before testing. Chipped wet ice used was made in a Hoshizaki icemaker.

### Data loggers

Electronic temperature data loggers (Omega OM-141 and CryoPak iMINI) were used to monitor and record temperatures during this study. Data loggers were verified by comparing their recorded temperatures to NIST traceable thermometers. The data loggers are equivalent to each other in terms of measuring temperatures. They were arbitrarily chosen based on their availability. A single data logger was placed in each cooler for each run and the placement was consistent for each run. Omega data loggers were set to record temperatures every minute, while CryoPak iMINI data loggers were set to record temperatures every 44 seconds. For cooler #16, recordings occurred every 55 seconds. Data was download using either the ELUSB software from Omega or the Console Plus software for CryoPak. Recorded temperature data was exported to a CSV file and analyzed in Microsoft Excel and GraphPad Prism.

### Ice and ice water cold time determination

An ice water mixture was made by mixing chipped ice with deionized water in a large container. The ice water mixture was then poured into the cooler and weighed to ensure it contained 5 kg for every 20 L internal volume. For chipped ice alone, we use added chipped ice directly to the cooler without the addition of water. In these cases, ice was poured into each cooler and weighed to ensure coolers contained a standardized starting weight of 5 kg ice water mixture for every 20 L of internal cooler volume. Temperatures were taken of the ice and ice water mixture with a NIST calibrated IR digital thermometer (Catalog 4483, Traceable Products). A data logger was then set inside a plastic secondary container (Saf-T-Pak, STP-300), sealed and set within the cooler with the coolant. All coolers were left for a minimum of four days at ambient temperature in a climate-controlled room. The cold time was calculated by finding the difference between the first recording at time *t =* 0 minutes and the time just before the internal cooler temperature reaches 6°C. Cold times were measured at least twice. A correlation between price and cold time hours was performed using Pearson’s Correlation.

### Cold pack cold time determination

The data logger was placed directly between two cold packs within cooler #13. For some experiments, the data logger was placed in a secondary container, which was then placed next to two cold packs within the cooler. When noted, the secondary container was pre-chilled by placing it in a -20°C freezer overnight. The data loggers recorded cold pack temperatures for 2 days within the cooler in an ambient temperature controlled room. Cost effectiveness was calculated by dividing the cold time hours by total weight (0.45 kg x number of water bottles), then by the cold pack price.

### Modeling of cold time based on mass of coolant

Frozen water bottles were placed inside cooler #14 (rotomolded cooler), cooler #10 (injection molded), and the PS foam cooler and left for 4 days in an ambient temperature controlled room. The cold time hours were then plotted against the number of frozen water bottles. Several models were fitted to determine the best fit in terms of reduced residuals and R^2^ values using GraphPad Prism.

### Statistical analysis

All statistics were calculated using GraphPad Prism version 6.07.

## Results

### Descriptive and physical analysis of the coolers

**Fig 1:**
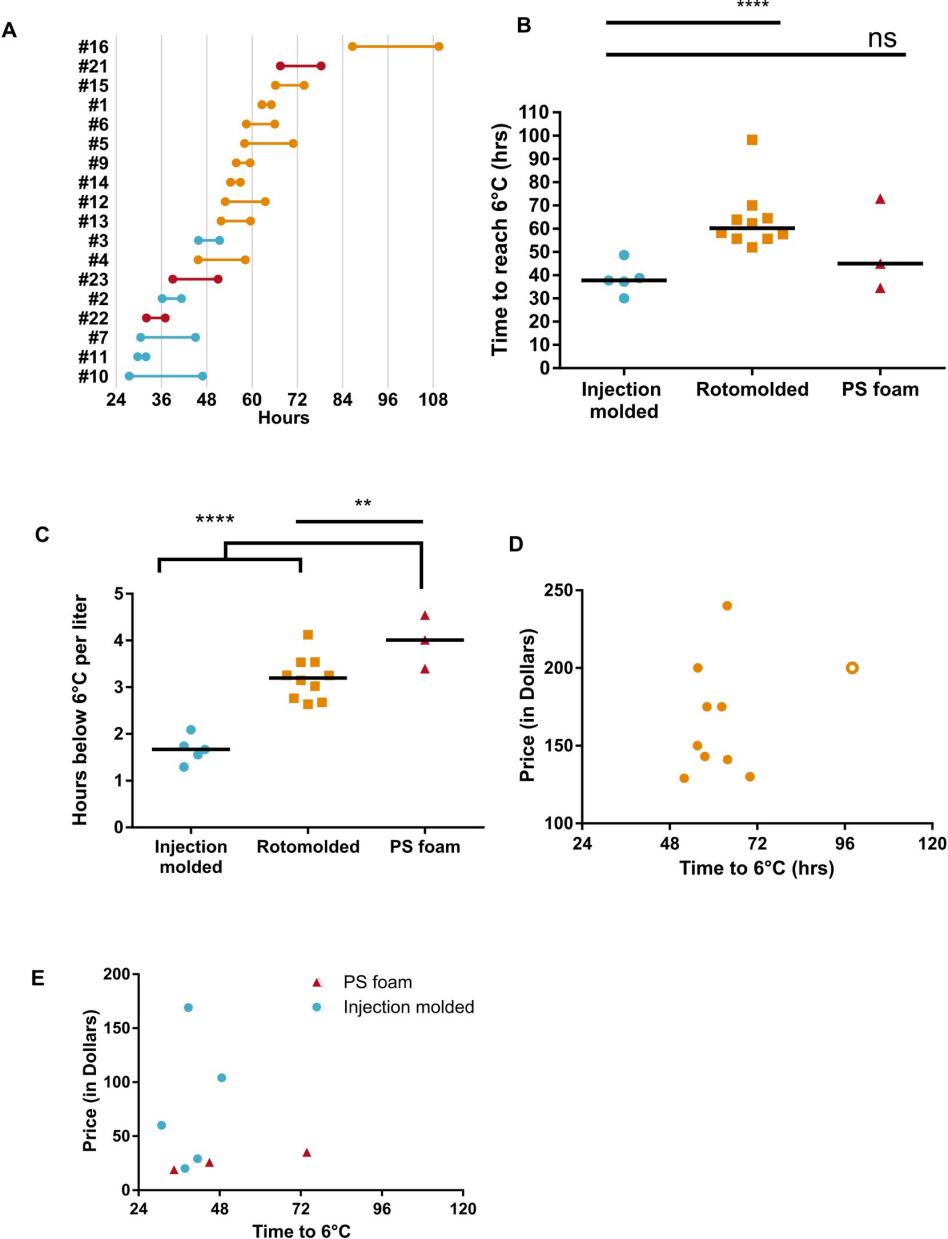
Comparison of different coolers’ time below 6°C. (A) Dumbell plot displaying the cold times for each cooler in the study. Points represent the time to reach 6°C and the line represents the range. (B) Average cold time in hours of coolers when 5 kg of ice-water is added per 20 L internal volume (or equivalent ratio). Each point represents the mean of 2 runs. Open symbols represent the largest coolers of each group. Bar represents the average. Statistical significance determined by a one-way ANOVA with Tukey’s multiple comparison test. ***P-value < 0.001 and ****P-value < 0.0001. (C) Cold time normalized to volume for each run of coolers. Each point represents the average run for each cooler type. Open symbols represent the largest internal volume coolers of each group. Bar represents the average. Statistical significance determined by a one-way ANOVA with Tukey’s multiple comparison test. ***P-value < 0.001 and ****P-value < 0.0001. (D) Scatterplot of rotomolded cooler price and cold time. Open symbol is cooler #16, while closed symbols represent all other rotomolded coolers. (E) Scatterplot of injection molded and PS foam cooler price and cold time. Blue circles are injection molded coolers and red triangles are PS foam coolers.

Eighteen coolers were purchased and their dimensions, weight, and miscellaneous qualities were analyzed (Table 1). All coolers had an internal volume between 8.7 L and 29.2 L. While most rotomolded and injection molded coolers were roughly 20 L or greater, it was noticed that the majority of PS foam coolers received at the CDC are smaller. Therefore, we included one PS foam cooler that was 20L and two PS foam coolers that are more typically used in shipping (8.7 L and 11.2 L). Rotomolded coolers were significantly heavier than injection molded and PS foam coolers (rotomolded mean: 7.1 kg (SD +/- 0.7 kg), injection molded mean: 3.0 kg (SD +/- 1.4 kg), PS foam mean: 0.82 kg (SD +/- 0.74 kg)). The ratio of weight to internal volume was also analyzed. Coolers with a low ratio could carry more and weigh less, while higher ratios have smaller capacities and weigh more. Injection molded coolers and PS foam cooler had lower ratio than rotomolded coolers (Table 1). The rotomolded coolers had a significantly higher price than the non-rotomolded coolers (rotomolded: $168.30 (SD +/- $36.47), injection molded: $69.50 (SD +/- $52.38), PS foam: $25.57 (SD +/- $8.09)). The external volume of rotomolded coolers were statistically larger than PS foam (rotomolded: 72.4 cm^3^ (SD +/- 18.6 cm^3^), injection molded: 50.6 cm^3^ (SD +/- 12.5 cm^3^), PS foam: 34.4 cm^3^ (SD +/- 26.6 cm^3^)). There was no statistical difference between the internal volume (Fig 1D). All rotomolded coolers had the ability to be secured by a padlock or zip tie and were leak proof due to an O-ring sealed lid. In contrast, many of the non-rotomolded coolers could not be locked or leaked water during the test. While the tight fitting O-ring seal on rotomolded coolers did prevent water leaking, it also necessitated that a vent be present for use with dry ice. One of the rotomolded coolers, #16 lacked a vent, precluding the use of dry ice.

**Table 1:** Description of coolers used in study.

Rotomolded coolers														
Cooler ID	6	16	14	12	4	9	1	5	15	13	Average	SD	P-value vs injection molded^a^	P-value vs PS foam^a^
Cost ($)	$ 175.00	$ 200.00	$ 150.00	$ 175.00	$ 129.00	$ 143.00	$ 141.00	$ 240.00	$ 130.00	$ 200.00	$ 168.30	$ 36.47	**	***
Empty Weight (kg)	6.5	8.2	6.6	7.3	7.5	6.0	8.1	7.3	6.5	7.2	7.1	0.669	****	****
Ext. Volume (L)	65.8	126.8	62.6	68.6	77.4	65.8	62.1	64.2	65.8	65.4	72.4	18.6	NS	*
Int. Volume (L)	17.7	27.7	18.4	22.1	19.4	17.7	23.1	19.6	17.7	17.7	20.1	3.12	NS	NS
Weight/internal volume (kg/L)	0.37	0.30	0.36	0.33	0.39	0.34	0.35	0.38	0.37	0.41	0.35	0.21	****	****
One person carry?	Yes	Yes	Yes	Yes	Yes	Yes	Yes	Yes	Yes	Yes				
Durable?	Yes	Yes	Yes	Yes	Yes	Yes	Yes	Yes	Yes	Yes				
Resistant to decontamination?	Yes	Yes	Yes	Yes	Yes	Yes	Yes	Yes	Yes	Yes				
Leak proof?	Yes	Yes	Yes	Yes	Yes	Yes	Yes	Yes	Yes	Yes				
Can stickers be applied?	Yes	Yes	Yes	Yes	Yes	Yes	Yes	Yes	Yes	Yes				
Can you mark on the coolers?	Yes	Yes	Yes	Yes	Yes	Yes	Yes	Yes	Yes	Yes				
Can coolers be padlocked?	Yes	Yes	Yes	Yes	Yes	Yes	Yes	Yes	Yes	Yes				
Availability?	Common	Common	Common	Common	Common	Common	Common	Common	Common	Common				
Dry ice ok?	Yes	No	Yes	Yes	Yes	Yes	Yes	Yes	Yes	Yes				
**Injectional Coolers**														
Cooler ID	7		11		3		2		10		**Average**		**SD**	**P-value vs PS Foam**^**a**^
Cost ($)	$ 20.00		$ 60.00		$ 104.00		$ 169.00		$ 29.00		$ 69.50		$ 52.38	NS
Empty Weight (kg)	2.3		2.5		4.1		5.7		2.0		3.0		1.4	*
Ext. Volume (L)	53.1		38.9		60.6		68.8		31.6		50.6		12.5	NS
Int. Volume (L)	29.2		17.7		29.1		18.6		23.9		23.7		4.5	NS
Weight/internal volume (kg/L)	0.080		0.14		0.14		0.31		0.081		0.15		0.083	NS
One person carry?	Yes		Yes		Yes		Yes		Yes					
Durable?	Yes		Yes		Yes		Yes		Yes					
Resistant to Decontamination?	Yes		Yes		Yes		Yes		Yes					
Leak proof?	No		Yes		No		Yes		No					
Can stickers be applied?	Yes		Yes		Yes		Yes		Yes					
Can you mark on the coolers?	Yes		Yes		Yes		Yes		Yes					
Can coolers be padlocked?	No		No		No		Yes		No					
Availability?	Plentiful		Common		Common		Common		Plentiful					
Dry ice ok?	Yes		Yes		Yes		Yes		Yes					
**Polystyrene foam cooler**														
Cooler ID	21		22		23		**Average**		SD					
Cost ($)	$ 35.00		$ 25.82		$ 18.88		$ 26.57		$ 8.09					
Empty Weight (kg)	1.7		0.24		0.56		0.82		0.739211					
Ext. Volume (L)	65.0		20.1		18		34.4		26.56379					
Int. Volume (L)	27.1		8.7		11.2		15.7		10.00133					
Weight/internal volume (kg/L)	1.7		0.24		0.56		0.82		0.739211					
One person carry?	Yes		Yes		Yes									
Durable?	No		No		No									
Resistant to Decontamination?	No		No		No									
Leak proof?	No		No		No									
Can stickers be applied?	Yes		Yes		Yes									
Can you mark on the coolers?	Yes		Yes		Yes									
Can coolers be padlocked?	No		No		No									
Availability?	Plentiful		Plentiful		Plentiful									
Dry ice ok?	Yes		Yes		Yes									

^a^ One Way ANOVA with Tukey's Multiple Comparison Test

* *P-*value <0.05

** *P*-value <0.01

***P-value <0.001

**** *P*-value <0.0001

### Rotomolded coolers and PS foam keep cold temperatures longer than injection-molded coolers

Insulated coolers are known to hold cold temperatures for extended periods of time, but it is possible that the amount of time varies by cooler type. To quantify the amount of time these types of coolers stay cold, we selected 18 different coolers: five injection molded, three PS foam, and 10 rotomolded coolers. We measured each cooler’s cold time, defined as the time a cooler could maintain temperatures below 6°C. This temperature was chosen since most refrigerated laboratory samples require storage temperatures between 2–8°C. All coolers were placed at ambient temperature inside a climate-controlled building (average 17.6°C, range: 13.4°C– 23.4°C, S1A Fig). For coolant, we used an ice-water mixture to hold at 0°C until all the ice melts to water. Due to issues with water leaking from PS foam coolers, the cooler was lined with a plastic bag before the ice-water slurry was added. Injection molded coolers warmed to 6°C quicker than all other cooler types, with an average of 38.62 hours (SD ± 8.53 hours, Fig 1A and 1B). The rotomolded coolers cold time was significantly longer than injection molded coolers, with an average of 63.72 hours (SD± 13.56 hours). The PS foam cooler’s cold time averaged 54.32 hours (SD ± 19.33 hours). Only one rotomolded cooler, #16, had a greater cold time than PS foam with 98.05 hours below 6°C. The difference in ambient temperature was found to lead to an increase in time below 6°C. Coolers #5, #6, #10, #13, and #15 were run on 16 October and 27 October, but the external temperature on 27 October was an average of 5.2°C colder. Cooler numbers #5 and #10 had an extra 12.6 and 19.4 hours below 6°C, respectively, on the colder run (Fig 1A). Coolers #6, #13, and #15 lasted an extra 7.5, 7.8, and 7.6 hours longer below 6°C, respectively, on the colder run. Because of the difference in volume between some coolers, particularly the PS foam, we normalized the cold time by the volume (Fig 1C). When normalized by internal volume, PS foam and rotomolded coolers were significantly greater than five coolers. This suggests that the coolers have similar insulation regardless of their size.

Next, we wanted to see if there were correlations between the price of rotomolded coolers and their performance. A Pearson’s Correlation analysis found the slope of the curve to be not significantly different from zero (Pearson’s Correlation = 0.303, *P*-value = 0.394; Fig 1D). Indeed, the least expensive 5 coolers (<$150) generally performed as well as the 5 most expensive. The sole exception was the cooler #16, which maintained cold the longest of all coolers with 98.1 hours below 6°C and was $200. Injection molded coolers similarly had no correlation between price and cold time (Fig 1E, blue circles). These coolers also varied more along the price axis than the cold time axis. PS foam coolers, though, showed very little change in price, but could vary dramatically in cold chain time (Fig 1E, red triangles).

### Block ice maintains cold temperatures longer than chipped ice or an ice-water slurry

**Fig 2:**
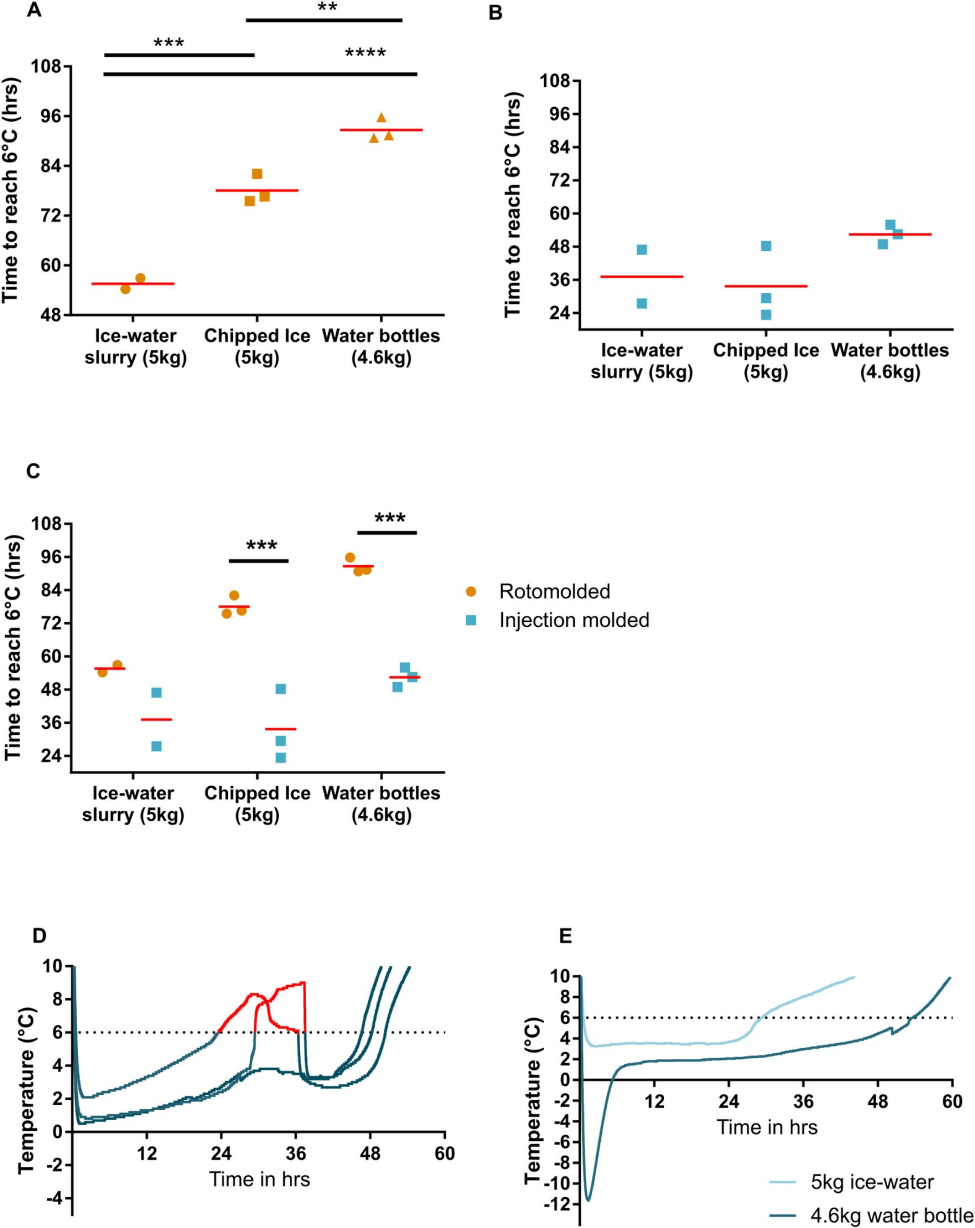
Different types of ice vary in their cold times. (A) Cold time for rotomolded cooler #14 with 5 kg of ice-water, chipped ice, and solid blocks of ice. Each point represents a single run and the red bar represents the mean. Statistical significance determined by a One-way ANOVA with Tukey’s multiple comparison test. **P-value ≤ 0.01, ***P-value ≤ 0.001, ****P-value ≤ 0.0001. (B) Cold time for injection molded cooler #10 with 5 kg of ice-water, chipped ice, and solid blocks of ice. Each point represents a single run and the red bar represents the mean. (C) Cold time for varying types of ice in rotomolded cooler #14 (orange) and injection molded cooler #10 (blue). Red bar represents the mean. Statistical significance determined by One-way ANOVA with Holm-Šidák multiple comparison test. ***P-value ≤ 0.001. (D) Temperature curves for injection molded cooler #10 in chipped ice. Dotted line is 6°C cutoff and red section of the line highlights time exceeding 6°C. (E) Temperature curves for injection molded cooler #10 with ice-water (light blue) and frozen water bottles (dark blue).

When used as a coolant, ice can be supplied as solid blocks or chipped. We tested whether these forms of ice differed in their cold time. To make blocks of ice, we froze 0.45 kg of water in a -20°C freezer overnight. We then chose rotomolded cooler #14 and injection molded cooler #10 for further testing. For the rotomolded coolers, replacing 5 kg ice-water slurry with 5 kg of chipped ice increased cold time by 22.5 hours (from 55.6 hours to 78.1 hours), while using 4.6 kg frozen water bottles increased cold time by 37 hours (from 55.6 to 92.6 hours) (Fig 2A). Injection molded coolers with 4.6kg frozen water bottles increased the cold time by 14.6 hours (Fig 2B). However, there was no increase in time between using an ice-water slurry and chipped ice. There were significant differences in the cold time between the rotomolded coolers and injection molded coolers with chipped ice and frozen water bottles (Fig 2C). It was noticed the injection molded cooler with chipped ice had unusual temperature curves compared to those with ice-water slurry or frozen water bottles (Fig 2D and 2E). In particular, two experiments with the injection molded cooler containing chipped ice exceeded the 6°C threshold for 8 and 13 hours before rapidly decreasing in temperature (Fig 2D, red lines). Therefore, a solid block of ice outperforms other forms of ice, but the amount of time gained varies depending on the cooler type.

### Cold packs vary in their cold time compared to frozen water bottles

**Fig 3:**
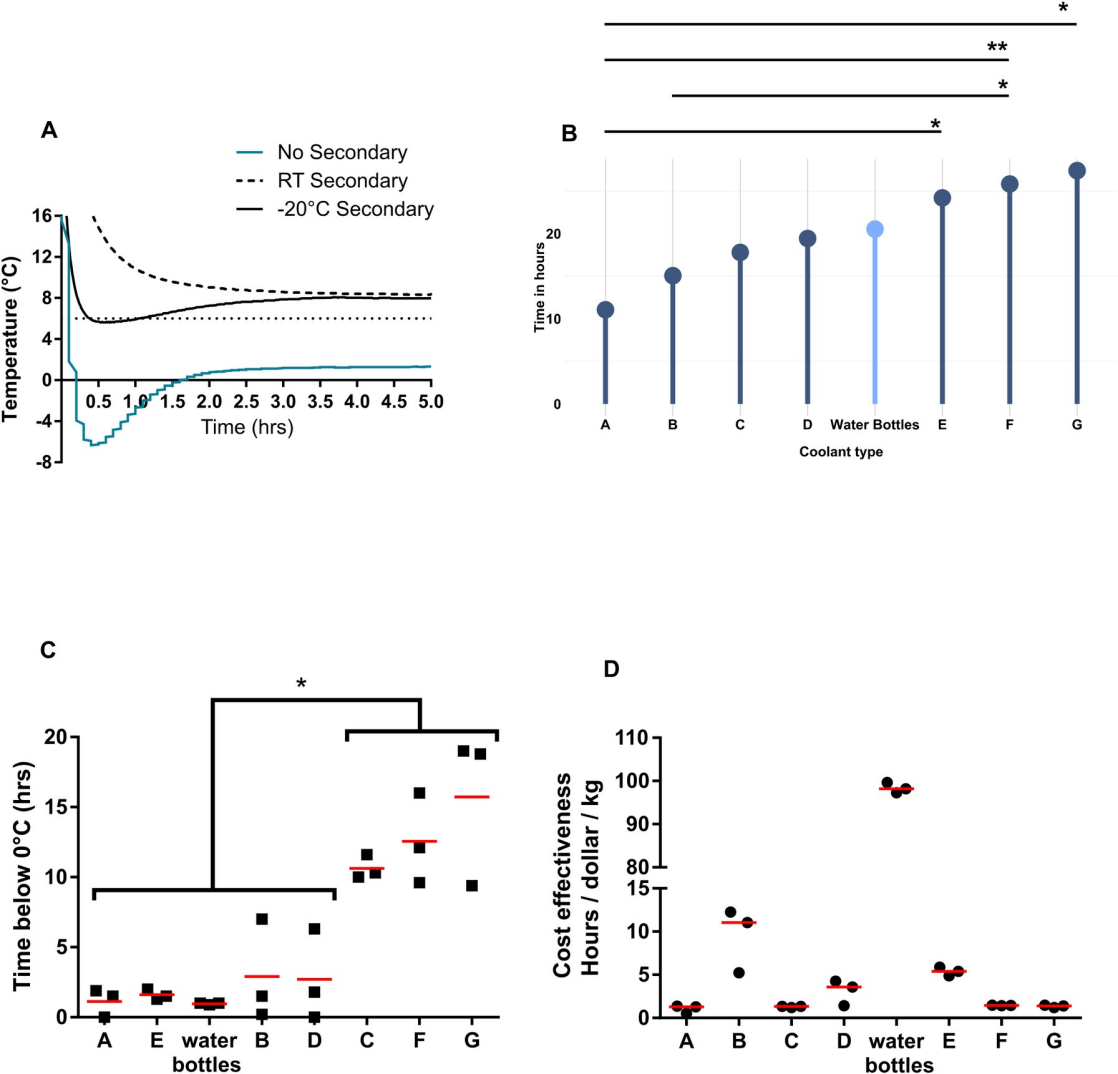
Comparison of cold packs and their cost efficiency. (A) Temperature curve in rotomolded cooler #13 with 0.45 kg of cold pack E with and without secondary containers. Lines represent the average of three experiments. (B) Cold time for various cold packs and the frozen water bottles in rotomolded cooler #13. Point represents the mean of three independent runs. Experiments were run in triplicate. Statistical significance was determined by a one way ANOVA with Tukey’s multiple comparison test. *P-value < 0.05, **P-value < 0.01. (C) Time in hours 1 kg of cold packs remained below freezing when not in a secondary container. Each point represents an independent run and the red bar represents the average. Statistical significance was determined by a one way ANOVA with Tukey’s multiple comparison test. *P-value < 0.05. (D) Cost effectiveness of cold packs as determined by cold time in Fig 3A. Red bar represents the mean, n = 3.

We next investigated how proprietary cold packs compared to frozen water bottles. Cold packs are available in various forms and prices, but essentially offer a re-usable way to maintain cold temperatures during shipment. We measured the cold time from seven commercially available cold packs along with frozen water bottles. We selected cooler #13 as it had a similar cold time as cooler #14, which was being used for experiments in Fig 2 (Fig 1A). One kilogram of the cold packs were placed in the same rotomolded cooler (#13) to standardize the amount of coolant and cooler. We initially measured the cold time with 1 kg of coolant with the data logger within a secondary container. Surprisingly, we noticed the 1 kg of Cold Pack E was not able to lower temperatures below 6°C (Fig 3A). We hypothesized that using a room temperature secondary container prevented the cold pack from reaching 6°C and repeated the run with a secondary container chilled overnight at -20°C. However, this only led to a brief cold time below 6°C for 39.6 minutes. We confirmed the cold packs themselves were able to remain below 6°C by placing the data logger next to the cold packs. In these experiments, the cold packs had an average cold time of 24.2 hours (Fig 3B). This suggested that the secondary container act as an insulator. Therefore, we continued our studies with all cold packs directly next to the data logger to understand how long they remain below 6°C irrespective of the secondary container.

The cold time varied from 11.1 hours to 27.4 hours for the cold packs (Fig 3B). Cold packs E, F, and G had significantly longer cold times than cold pack A (One-Way ANOVA with Tukey’s Multiple comparison test, *P-value <0.05 and **P-value <0.01). Cold pack G also had a significantly longer cold time than cold pack B (One-Way ANOVA with Tukey’s Multiple comparison test, *P-value <0.05). Frozen water bottles’ cold time was close to the median cold time of all cold packs (water bottle median cold time = 20.5 vs. median cold time of all cold packs = 20.6).

All cold packs had temperatures drop below 0°C. Cold packs C, F, and G were below 0°C for the longest average time with 10.8, 12.9, and 13.3 hours, respectively (Fig 3C). This was an average of 56%, 52% and 47% of the entire time below 6°C. Their minimum temperatures were all below -10°C. On the other side of the spectrum, water bottles had the highest average minimum temperature at -2.1°C and the shortest amount of time below 0°C at 1 hour. Five kilograms of ice frozen in water bottles, however, were below freezing for 5.2 hours (SD ± 0.52 hours) with an average minimum of -11.7°C (SD ± 0.95°C) (Fig 2E). There was also a large amount of variability in the 4 of the 8 cold packs. Cold packs B, D, F, and G showed large differences in the time below freezing (Fig 3C). Analysis of the initial cold pack temperature, room temperature, and freezer temperature varied minimally during these runs (Figs 3D and S1B).

While the water bottle cold time was not as long as Cold Pack G, they were more cost effective. We defined cost effectiveness as the amount of cold time per dollar per kilogram for each cold pack and water bottle. Most cold packs were between 1–5 hours/dollar/kg (Fig 3D). Cold pack B had an average of 9 hours/dollar/kg, while frozen water bottles had an average of 98 hours/dollar/kg. The water bottle calculation assumes the water bottles were purchased, rather than recycled, but using recycled or discarded water bottles would lead to higher cost effectiveness. This suggests that the addition of a few water bottles would lead to similar cold times as the best performing cold pack for a minimal addition of cost.

### Modeling cold time based on cooler type and weight

**Fig 4:**
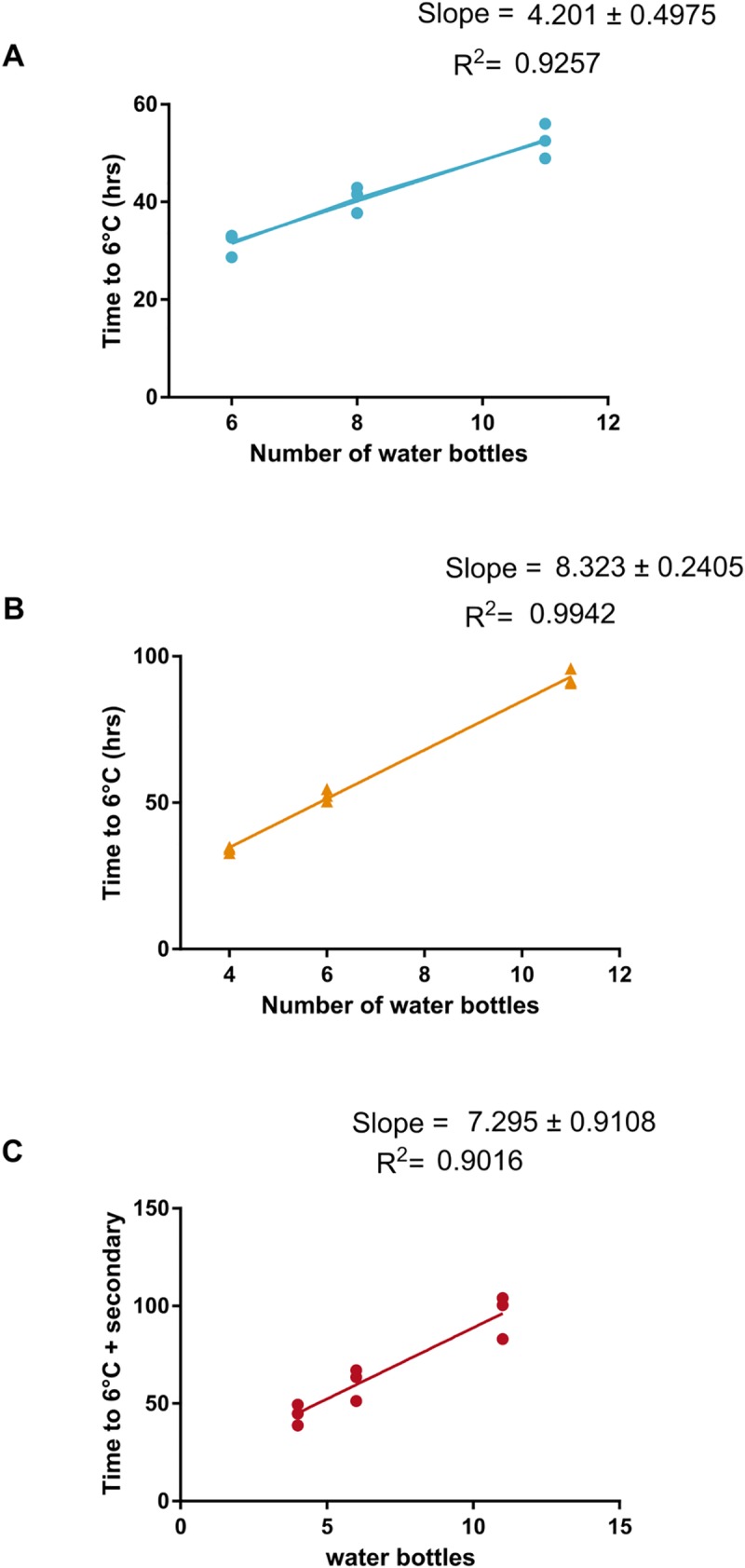
Modeling cold time as a function of the amount coolant. Cold time was plotted as a function the amount of frozen water bottles and a linear function was fitted in (A) Injection molded coolers, (B) rotomolded coolers, and (C) PS foam coolers (n = 3 for all coolers). The R^2^ was calculated for each set of coolers.

We hypothesized that cold time is in part dependent on the amount of coolant and type of cooler. To test this, we varied the amount of coolant we added to a rotomolded, PS foam or injection molded cooler and measured the cold time. Since the type of coolant affected the cold time, we chose frozen water bottles as they were close to the median cold time of all cold packs and it was easy to acquire large numbers of water bottles (Fig 3B). For all coolers, we found a linear model to be the best fit. For the injection-molded coolers, we used six, eight, and 11 water bottles, corresponding to 2.7 kg, 3.6 kg and 5 kg, respectively. A linear model gave an R^2^ of 0.9257 a slope of 4.2 hours/kg (Fig 4A). The rotomolded coolers were tested with four (1.8 kg), six, and eleven water bottles. The linear model had an R^2^ value of 0.99, a slope of 8.3 hours/kg (Fig 4B). Finally, the PS foam coolers were very similar to the rotomolded coolers. The linear model gave an R^2^ of 0.90 and a slope of 7.3 hours/kg (Fig 4C).

## Discussion and conclusions

Maintaining specimens at the appropriate temperature is critical for laboratory sample integrity and testing results, particularly when cold temperatures are required [2, 6, 11]. Currently, polystyrene foam and injection molded plastic coolers are primarily being used in laboratory specimen transport systems. But as rotomolded coolers become more popular within the community and cost of these coolers decrease over time, we expect greater numbers to be used in the field to strengthen specimen transport networks. Understanding how these coolers perform and how differing coolants affect their cold chain duration time is critical in planning specimen transport networks, which will ensure samples can arrive to testing laboratories in good condition. In this study, polystyrene foam, injection molded coolers, and rotomolded coolers were evaluated to determine how long they kept temperatures below 6°C based on different types and amounts of coolants.

Our results demonstrate that rotomolded coolers and polystyrene foam coolers remained below 6°C significantly longer than injection-molded coolers. Polystyrene foam remained cold for longer than almost all other types of coolers. These coolers are inexpensive and can also be repurposed to IATA-approved shippers for transportation through private carriers [12–14]. However, the PS foam coolers consistently leaked water during our procedures. Furthermore, polystyrene foam coolers are less durable than plastic coolers and will need to be replaced more often, which is potentially problematic in areas with procurement issues. PS foam is both porous and a hydrocarbon polymer. As such, it may be challenging to decontaminate PS foam as many alcohol-based disinfectants could damage the insulation. More research in to the decontamination of PS foam would allow for better guidelines in their reusability involving transport of infectious material. Rotational molded coolers are the most expensive coolers in our study, but have similar cold times as polystyrene foam and they can be decontaminated after each use. This allows them to be reused multiple times and beneficial in creating a long-term sustainable specimen transport system.

We also found that the span of performance for the cold packs ranged 12 hours. Frozen water bottles were closest to the median of all the cold packs. While several cold packs stayed below 6°C longer than frozen water bottles, they also were below 0°C for prolonged periods of time. This can be problematic as cold chain temperature requirements can vary based on different applications. The research described suggests that freezing and going below cold chain requirements occurs often but is undetected unless a datalogger is included. Certain vaccines, immune globulin and whole blood should not be frozen or their integrity will be compromised. In fact, vaccines were found to be below the recommend threshold in 21% of shipments in lower income countries and 38% of shipments in wealthier countries [10]. Even if a datalogger is included, we noted significant variability in the times below freezing for several cold packs. Four of the eight cold packs (B, D, F and G) had significant variability in their times below 0°C with minimal changes in freezer temperature, initial cold pack temperature, or room temperature. When such variability occurs in a highly controlled laboratory space, it will likely occur in the field. Therefore, coolers holding freeze-sensitive products should avoid coolants that are stored at -80°C or -20°C or allow for several hours for the coolant and cooler to reach 2–8°C. Future studies could also examine the feasibility and model using less cold (2–8°C) coolant that is replenished often. Regardless, the data suggest that careful analysis of cold chain coolants is required.

The type of ice was found to have an effect on cooling in rotomolded coolers, but not injection molded coolers. Ice that was frozen in a large contiguous block, such as in a frozen water bottle, had substantially longer cold times than chipped ice or ice-water mixtures. Ice-water mixtures had the shortest cold time, as heat from the water is more easily transferred to ice. Interestingly, we found that chipped ice in an injection molded cooler at times exceeded the 6°C cutoff and then dropped back below to 6°C. In the beginning of these experiments, chipped ice would be supporting the secondary container and data logger. As time passed and the ice melted, there was less ice to cool the secondary container. Further, the secondary container was supported by an increasing unstable pillar of chipped ice. Eventually the container would crash into the melted ice water and result in a rapid cooling below the 6°C threshold. These results suggest that coolant choice can have unintended consequences, misleading the recipient laboratory on the quality of samples.

Frozen water bottles are also the most cost effective option. Plastic water bottles are ubiquitous and are a significant source of waste [15]. As such, it is feasible that they could be found for free in most locations. They are also rugged enough to be used multiple times and easily decontaminated between uses. Finally, in contrast to using chipped ice, they can be easily charged or recharged within a freezer allowing cooler packing and unpacking to be quickly performed.

By varying the number of water bottles, we were able to build a predictive model for how long different coolers will stay below 6°C. Predictive modeling can vary from relatively simple linear regression to advanced machine learning techniques such as support vector machines and neural networks. However, the simplest model that describes the data is often the best choice [16]. For all coolers, we found a linear model fit the data with high R^2^ values and small residuals. The slopes for each curve also provide a useful reference point to understand how much additional cold time can be achieved by adding additional frozen water bottles. PS foam and rotomolded coolers can add between 7–8 hours of cold time with the addition of a single water bottle, while the injection molded coolers only add 4.6 hours per additional water bottle. Importantly, the addition of water bottles assumes the bottle contains 0.45 kg of frozen water. A different size water bottle with a different amount of frozen water may have a different slope. The 0.45kg amount was used because it nearly filled the 0.5L water bottle, froze quickly, and could fit in almost any size cooler. This data provides a useful guideline for planning transportation routes based on the ability to maintain sample cold chain. In particular, these guidelines would be helpful in areas where there is standardization of shipping containers, materials and transportation [9].

There are, however, some limitations to these findings. The testing was performed in climate control rooms that kept a consistent temperature. It is expected that the cold chain performance will vary with external temperatures. On October 27^th^, a run occurred when the external temperature dropped by 5.2°C; this led to an increase in cold times for the coolers. However, these changes did not change the overall trend of rotomolded coolers staying colder than injection molded coolers. Indeed, the consistent trend suggests the data is valid even when variability occurs. All injection molded and rotomolded coolers were within a certain volume range and therefore, it is unknown if coolers of different sizes will perform similarly. The PS foam coolers did vary in sizes and showed a decrease in cold time based on size. While the difference was lost when normalized by internal volume, it does suggest that smaller coolers will need more coolant to last as long as 20L coolers. Additionally, our studies only analyzed how long the system maintained a refrigeration environment (2˚-6˚C) and not a frozen environment. Therefore, we cannot predict if the coolers have the same advantages when using dry ice or cold packs designed for very low temperatures.

Given the difficulty and frequent need for frozen samples, ongoing studies are examining how long these coolers maintain temperatures below -20°C. Another useful comparison would be to analyze the cold time for advanced cooler systems specifically designed for keeping medical samples cold. These coolers are designed to maintain temperatures at 4°C for over 72 hours, but their container size and cost are much larger than rotomolded coolers.

This study provides data that allows researchers and health workers to compare how long different types of coolers can keep samples below 6°C. It allows for the ability to know how long a transport cooler can maintain cold chain during transportation times. By comparing differing coolers, coolants, and amounts of coolers, we built a predictive model that can be used to plan specimen transport activities to ensure sample integrity during its transport.

## Supporting information

S1 Fig (TIF)

S1 Raw data (ZIP)
