# Supplementary figures and images for: Analysis and modeling of coolants and coolers for specimen transportation
Source: PLoS One. 2020 Apr 17;15(4):e0231093. doi: 10.1371/journal.pone.0231093 (PMC7164660; doi:10.1371/journal.pone.0231093)

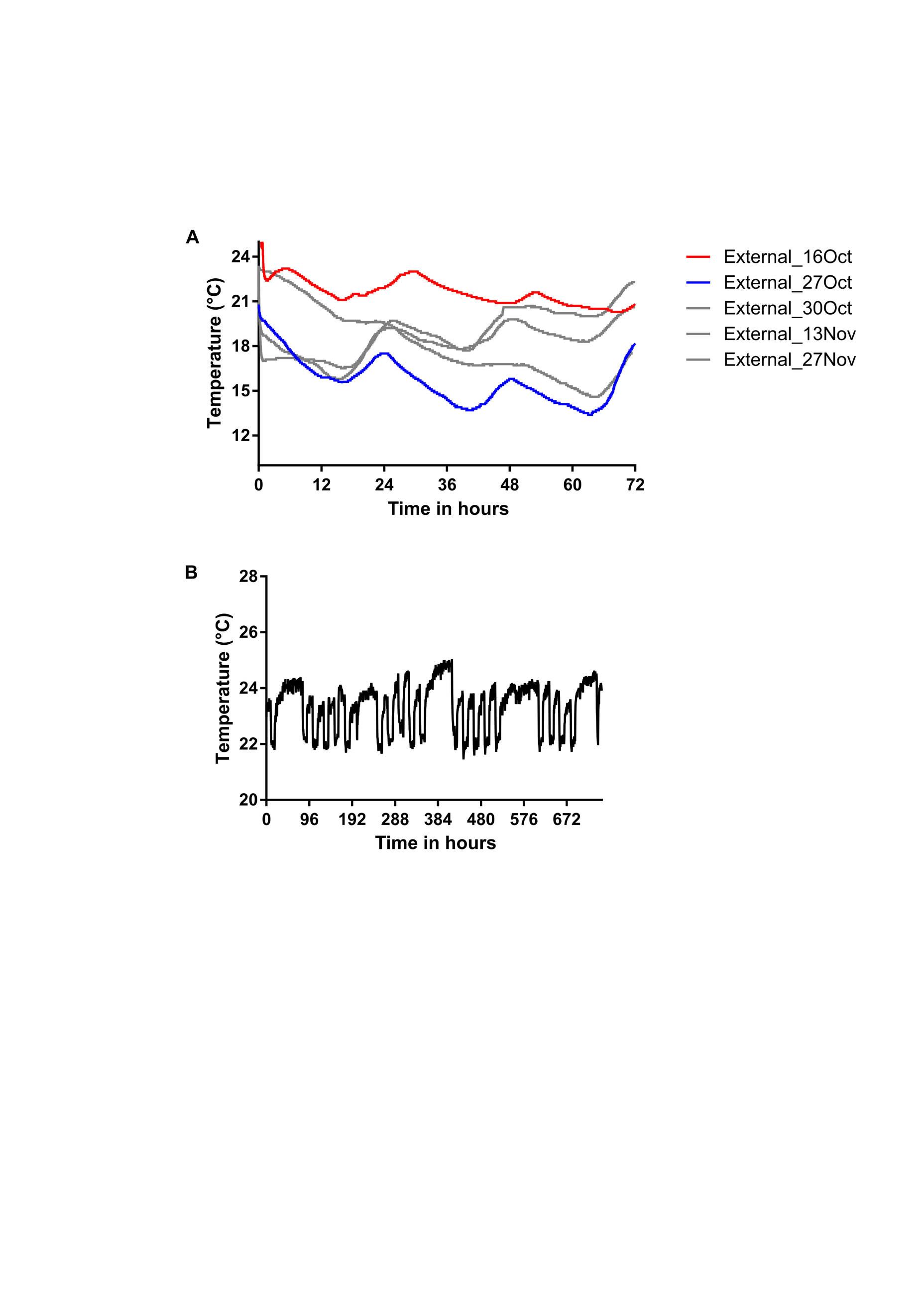

Supplement: S1 Fig — (TIF) [file pone.0231093.s001.tif]
